# Effect of spray-dried porcine plasma in peripartum sow feed on subsequent litter size

**DOI:** 10.1186/s40813-020-00180-0

**Published:** 2021-01-11

**Authors:** Joe Crenshaw, Laura Lafoz del Río, Luis Sanjoaquin, Simon Tibble, Francesc González-Solé, David Solà-Oriol, Carmen Rodriguez, Joy Campbell, Javier Polo

**Affiliations:** 1 APC LLC, 2425 SE Oak Tree Court, Ankeny, IA 50021 USA; 2 ThinkinPig, Avda. Gómez Laguna 41, 50009 Zaragoza, Spain; 3 Alternative Swine Nutrition (ASN). PL Fraga C/ Comunidad de Murcia parc. LIE 1-03, 22520 Fraga, Spain; 4 Animal Nutrition and Welfare Service (SNIBA), Department of Animal and Food Science, Universitat Autonòma de Barcelona, 08193 Bellaterra, Spain; 5 APC EUROPE, S.L. Avda, Sant Julià 246-258, Pol. Ind. El Congost, E-08403 Granollers, Spain

**Keywords:** Cytokines, Litter size, Oxidation status, Parturition, Sows, Spray-dried porcine plasma

## Abstract

**Background:**

Nutritional strategies for sows designed to reduce peripartum stress are suggested to support postpartum recovery and productivity. Spray-dried plasma (SDP) in sow feed has been reported to benefit sow and litter performance. Stressed animals fed diets with SDP have a more efficient immune response supporting animal recovery and health. The objectives of the present study using 452 sows (147 parity 1 sows, 148 parity 2 sows) were to determine if 0, 0.5 or 2.5% spray-dried porcine plasma (SDPP) in peripartum feed provided from entry in maternity through day 5 of lactation affects sow productivity and serological immune and oxidation status markers around parturition. Post-weaning sow productivity parameters including litter size at the next parturition was evaluated, but peripartum diets were only provided during the first parturition.

**Results:**

In the first parturition, total born litter size was lower (*P* <  0.05) especially for sows allotted to the peripartum diet with 2.5% SDPP. Percentage of stillborn pigs decreased quadratically (*P* <  0.05) for sows fed 0.5% or 2.5% SDPP compared to 0% SDPP in peripartum feed and this result was not affected by total born litter size. Serum glutathione peroxidase activity linearly increased (*P* <  0.01) with increased dietary SDPP for both prepartum and postpartum sampling periods. In the next parturition, total born pigs from combined data of parity 1 and 2 sows linearly increased (*P* <  0.05) and live born pigs tended (*P* = 0.09) to linearly increase as level of SDPP increased and this result was not affected by total born litter size in the first parturition. The change in total and live born pigs from the first to the next parturition linearly (*P* <  0.01) increased as dietary SDPP increased for parity 1 and 2 sows.

**Conclusions:**

The reduced percentage of stillborn pigs and increased litter size of parity 1 and 2 sows in the next parturition was independent of total born litter size in the first parturition suggesting SDPP in peripartum sow feed may have merit for reducing stillborn pigs and benefit litter size in the next parturition for parity 1 and parity 2 sows.

## Background

Most sow farms typically use a single gestation feed from breeding to entry in maternity followed by a single lactation feed from entry in maternity to weaning to accommodate sow movement and management of feed delivery and storage capacity. Litter size has increased steadily over the past several years and the nutritional needs for high-prolific sows increases, especially during late gestation to support the rapidly growing fetuses and to maintain sow body condition in preparation for lactation. In commercial production, daily gestation feed is typically restricted, and the amount provided is adjusted to control individual sow body condition. Bump feeding or simply increasing the daily allotted amount of gestation feed during late gestation has been commonly used to provide more daily nutrient intake to support sow body condition and the rapidly growing foeti. The benefits of bump feeding can be variable in commercial practice, but most report increased sow weight and a modest increase in pig birth weight, but an inconsistent effect on pig survival [1]. Amino acid, energy concentration, and (or) daily feed allowance in late gestation for sows with large litter size need to be adjusted to maintain sow body condition and support fetal growth during late gestation and throughout lactation [2–7]. Also, other studies have demonstrated benefits from using ingredients with high fiber content in gestation and lactation feed to support satiety, sow body condition and physiology, colostrum production, and survival of progeny [8–11].

Parturition is often prolonged with larger litter size which drains sow energy during labor and can lead to increased stillbirths, constipation, mastitis, metritis, and agalactia that negatively affects postpartum uterine recovery, and subsequent milk production, progeny survival and growth [12–16]. A peripartum or transition feed should be considered to provide nutrients and ingredients that address needs for the prolific sow in late gestation to help mitigate early postpartum stress and prepare the sow for ad libitum consumption of lactation feed to support milk production, progeny growth and survival, and subsequent sow reproductive performance. This targeted application of peripartum feed should be provided to sows as they are moved to maternity and continued for a few days after parturition to facilitate the management and logistics associated with sow movement, feed delivery and storage at the maternity facility.

Spray dried animal plasma (SDP) of porcine (SDPP) or bovine origin (SDBP) has been used in nursery pig diets because of its benefits on post-weaning growth, feed intake, morbidity, and survival [17, 18]. Sows fed SDP in lactation feed had several improvements in productivity measures including higher feed intake for young sows, heavier litter and average pig weight at weaning, improved pig survival, reduced wean to estrus interval, and improved farrowing rate to the next litter [19–23]. Spray dried plasma contains a diverse mixture of components including albumen, globulins, peptides, growth factors, and other components [18]. Research primarily using mice demonstrates that SDP supplementation in feed modulates the efficiency of the common immune systems, including the gastrointestinal, [24–26] respiratory, [27, 28] and reproductive systems, [29, 30] and restores homeostasis of lymphocytes Th-1 and Th-2 balance [25] under an activated immune response initiated by various types of pathogen or environmental stress. One study [26] using weaned pigs has demonstrated 5% SDP in feed reduced diarrhea, intestinal permeability, and inflammation in intestinal tissue. More specifically, transport stressed pregnant mice fed diets with 1, 2, 4 or 8% SDP maintained higher pregnancy rates compared to control mice. Only 1 day after feeding transport stressed pregnant mice diets with 1 or 8% SDP, there was a rapid restoration of Th-1/Th-2 balance in uterine tissue compared to control mice, which had elevated pro-inflammatory cytokines for a longer duration in early pregnancy [30]. In late pregnancy, 8% dietary SDP attenuated inflammation in uterine and placenta tissue and reduced lethargic effects induced by injected lipopolysaccharide [29].

The objectives of the present study were: 1) to determine if titrated levels of 0, 0.5% or 2.5% SDPP formulated in peripartum feed affected sow productivity and serology parameters around the initial parturition and 2) to determine if there were long-term effects from providing peripartum feed only during the initial parturition on post-weaning sow productivity parameters including litter size of the next parturition.

## Materials and methods

### Animal care statement

The experiment was done at a commercial sow farm that fulfilled animal housing standards and management by farm animal caretakers, trained to abide by authoritative animal welfare standards established by the *Law on Animal Welfare 2/2008* published by Diari Oficial de la Generalitat Catalunya [31].

### Animals and housing

The study was conducted at a commercial farm in Spain with 2000 sows. Maternity rooms were mechanically ventilated with high-flow fans and individual room temperature control. There were 27 maternity rooms, each with 16 crates. Each crate had individual sow feeders equipped with feed drop tubes and drinkers and supplemental heat pads for the piglets. At this farm sows were farrowed naturally. There were 452 Naïma Choice Genetics sows (average parity 2.63 ± 0.08; parity 1 to 8) used for the study that had their initial parturition from November 2018 through March 2019 and the average age of pigs at weaning was 24.3 ± 0.23 d. Sows were managed using a continuous flow through maternity rooms, thus requiring a rolling random allotment of sows to peripartum diets with an attempt to balance diet allotment as evenly as possible across sow parity as they entered maternity. Table 1 shows the number of sows per parity and diet after all sows had been allotted in the study.

**Table 1:** Number of sows per diet, parity, and sow group during the initial and next parturition

	Parity and number of sows at initial parturition^a^								All^b^	Y^c^	M^d^
Diet^e^	1	2	3	4	5	6	7	8	1–8	1–2	3–8
0	47	47	11	10	14	16	2	0	147	94	53
0.5	55	56	9	17	15	7	6	2	167	111	56
2.5	45	45	11	12	14	5	6	0	138	90	48
Sum	147	148	31	39	43	28	14	2	452	295	157
	Parity and number of sows at next parturition^f^								All	Y	M
Diet	1	2	3	4	5	6	7	8	1–8	1–2	3–8
0	45	42	11	10	13	15	2	0	138	87	51
0.5	50	53	9	15	14	7	5	2	155	103	52
2.5	43	42	11	12	14	5	6	0	133	85	48
Sum	138	137	31	37	41	27	13	2	426	275	151

^a^ Sow parity during initial parturition. Parity 1 is defined as a gilt having her first litter

^b^ All sows regardless of parity were used for group All statistical analysis

^c^ Only parity 1 and 2 sows were used for group Y (young sows) statistical analysis

^d^ Only parity 3 to 8 sows were used for group M (mature sows) statistical analysis

^e^ Diet is peripartum dietary treatment containing 0, 0.5 or 2.5% spray dried porcine plasma that was fed only during the initial parturition from entry in maternity through day 5 of lactation

^f^ Original sow parity at initial parturition and number of sows that had their next parturition

Standard production parameters, including litter size of total pigs born, pigs born alive, stillborn pigs, mummified pigs, pigs after cross-fostering, and pigs weaned were recorded for the initial parturition. According to the study protocol, cross-fostering of litters during the initial parturition was only to be done within 48 h postpartum and only within room and within dietary treatment. However, cross-fostering was not successfully accomplished as requested due to the difficulty to manage piglet welfare yet maintain litter integrity within treatment groups throughout the lactation period at this highly prolific sow farm. Therefore, progeny data after cross-fostering to weaning was excluded from the final data analyses due to these uncontrollable, but confounding factors that make it difficult to manage sow studies, especially when having more than two dietary treatments.

Other parameters evaluated included ultrasonic backfat depth of sows at the day of entry to maternity and at weaning, sow mortality and culls prior to movement to breeding, percentage of sows moved to breeding, the intervals for wean-to-first-estrus (date of first estrus minus date of weaning) and wean-to-final-service (date of final service minus date of weaning), percentage of sows completing their next parturition, and litter size at birth of the next parturition. In addition the change in total and live born litter size from the initial to next parturition was calculated, considering total born litter size of the initial parturition was established before peripartum diets were fed. Post-weaning sow reproductive performance and litter size information at birth for the next parturition was recorded to determine if prior feeding of the peripartum feed in the initial parturition impacted subsequent litter size in the next parturition.

### Experimental peripartum sow diets

Three peripartum sow diets containing either 0, 0.5 or 2.5% SDPP were formulated to provide similar levels of net energy and amino acids. Soybean meal was the primary protein source replaced by 2.5% SDPP (Table 2). Sows were provided 3 kg/d of their assigned peripartum feed pellets from the day of entry in maternity to the day of parturition (average 6.1 days), and 4.5 kg/d from day 1 to 5 of lactation. Peripartum feed output and refusals were recorded during the peripartum period. Average days of the peripartum period and average daily feed intake per sow corrected for feed refusals are presented in Table 3. Sows were provided a common lactation feed for the remainder of the lactation period to weaning and common breeding-gestation feed to the next parturition, however feed consumption per sow was not recorded during these production periods. The standard feeding practice for lactating sows at this farm was to gradually increase feed to ad libitum intake by day 7 of lactation and continue providing feed ad libitum to weaning. Lactation feed was provided 3 times per day to provide ad libitum intake. During the first month of pregnancy, sows in good body condition at weaning were provided 2.2 to 2.4 kg of gestation feed per day, while thin sows were provided 3.0 to 3.5 kg per day. After the first month of pregnancy, all sows were provided 2.4 kg of gestation feed per day. The gestation feed was provided once daily.

**Table 2:** Ingredient and nutrient composition of peripartum sow diets

Ingredient, %	0% SDPP	0.5% SDPP	2.5% SDPP
Barley	31.61	30.77	30.35
Corn	15.00	15.00	15.00
Wheat	10.00	10.00	14.00
Wheat bran	20.00	20.00	20.00
Soybean meal (47% CP)	10.30	10.70	5.70
Spray-dried porcine plasma^a^	0.00	0.50	2.50
Ligno-cellulose product^b^	2.50	2.50	2.50
Beet pulp	7.50	7.50	7.50
Soy oil	0.60	0.63	0.20
Calcium carbonate	1.65	1.70	1.70
Monocalcium phosphate	0.04	0.00	0.00
Salt	0.44	0.40	0.25
L-lysine HCL	0.05	0.00	0.00
L-threonine	0.015	0.00	0.00
VTM premix^c^	0.30	0.30	0.30
Analyzed nutrients			
Dry matter, %	87.2	87.3	87.9
Crude protein, %	14.4	15.2	14.6
Calculated net energy, kcal/kg	2101	2100	2100
Calcium, %	0.90	1.04	1.03
Phosphorus, %	0.43	0.45	0.46
Calculated digestible phosphorus, %	0.25	0.25	0.25
Lysine, %	0.65	0.67	0.69
Methionine, %	0.23	0.22	0.22
Methionine + Cysteine, %	0.44	0.44	0.47
Threonine, %	0.56	0.53	0.57
Tryptophan, %	0.19	0.20	0.19
Isoleucine, %	0.59	0.61	0.57
Valine, %	0.64	0.68	0.68

^a^ APPETEIN GS, APC Europe S.L., Granollers, Spain

^b^ FibreCell 5, Agromed Austria GmbH, Kremsmünster, Austria. Ligno-cellulose product made from wood

^c^ ASN GESTATING SOWS 3 FIT; a commercial vitamin-trace mineral premix plus phytase. Provided the following per kg: vitamin A, 8500 UI; vitamin D3, 1500 UI; vitamin E, 30 total; vitamin E, 15 UI; moliphenol, 3 g, vitamin K3, 2 g; vitamin B1, 1.5 g; vitamin B2, 3 g; vitamin B6, 2 g; vitamin B12, 20 mg; pantothenic acid, 10 g; niacin, 15 g; folic acid, 2 g; biotin, 200 mg; choline, 300 g; Fe, 80 g; Cu, 10 g; Zn, 90 g; Mn, 60 g; selenium total, 0.25 g; organic selenium, 0.1; iodine, 2 g; phytase 750

**Table 3:** Peripartum feed intake, backfat and postweaning reproductive performance of sows fed peripartum diets

		Peripartum diet, % SDPP^b^				Statistics (F-test, *P =*)^c^				
Variable	Group^a^	0	0.5	2.5	SEM	PR	D	L	Q	INT
Sows, n	All	147	167	138	–	–	–	–	–	–
	Y	94	111	90	–	–	–	–	–	–
	M	53	56	48	–	–	–	–	–	–
Entry to farrow days	All	5.95	6.42	6.11	0.19	0.03	0.17	0.97	0.06	0.03
	Y	5.46	6.02	6.29	0.25	0.31	0.05	0.04	0.19	0.24
	M	6.43	6.89	5.93	0.30	0.39	0.03	0.06	0.10	0.18
Total days diets fed	All	10.95	11.42	11.11	0.19	0.03	0.17	0.97	0.06	0.03
	Y	10.46	11.02	11.29	0.25	0.31	0.05	0.04	0.19	0.24
	M	11.43	11.89	10.93	0.30	0.39	0.03	0.06	0.10	0.18
Feed intake, kg/d^d^	All	3.77	3.76	3.77	0.09	< 0.01	0.67	0.70	0.42	0.25
	Y	3.76	3.75	3.76	0.10	0.24	0.32	0.56	0.17	0.14
	M	3.68	3.64	3.70	0.02	0.45	0.02	0.09	0.06	0.14
Entry backfat, mm	All	13.91	13.38	13.46	0.30	0.67	0.37	0.46	0.23	0.16
	Y	13.53	13.75	13.23	0.35	< 0.01	0.55	0.39	0.51	0.22
	M	13.39	13.04	13.62	0.60	0.09	0.71	0.61	0.56	0.39
Wean backfat, mm^e^	All	10.33	9.82	10.26	0.24	< 0.01	0.23	0.74	0.09	0.24
	Y	9.43	9.51	9.50	0.30	< 0.01	0.97	0.89	0.84	0.16
	M	10.65	10.17	10.89	0.53	0.38	0.49	0.50	0.37	0.58
Backfat loss, mm^e^	All	−3.73	−3.56	−3.36	0.31	< 0.01	0.48	0.25	0.73	0.86
	Y	−4.04	−4.02	−3.67	0.37	0.04	0.57	0.29	0.88	0.48
	M	−2.79	−2.95	−2.78	0.44	0.10	0.91	0.88	0.71	0.51
Sows to breeding, %	All	94.4	92.8	97.8	2.05	0.23	0.20	0.12	0.37	0.69
	Y	92.6	92.8	95.6	2.60	0.76	0.65	0.36	0.90	0.34
	M	97.3	93.8	100.0	3.55	0.88	0.32	0.34	0.32	0.87
Wean-to-first-estrus, d	All	8.63	8.65	8.96	0.64	0.92	0.92	0.68	0.96	0.36
	Y	8.47	8.04	9.59	0.84	0.15	0.38	0.22	0.53	0.26
	M	9.83	8.96	8.51	0.98	0.23	0.59	0.34	0.59	0.03
Wean-to-final-service, d	All	11.43	10.97	10.69	1.00	0.88	0.87	0.64	0.80	0.76
	Y	10.77	10.84	11.14	1.27	0.23	0.98	0.82	0.99	0.03
	M	12.63	10.86	10.02	1.73	0.53	0.51	0.30	0.53	0.52

^a^ Results for group All included data of all sows, group Y included data only for young (parity 1 and parity 2) sows and group M included data only for mature sows (parity 3 to 8)

^b^ Values are least squares means (LSM) and the pooled standard error of the mean (SEM) for peripartum diets by sow group

^c^ Each sow group was analyzed using a mixed model for the effects of PR (sow parity), D (peripartum diet), L (linear contrast of % SDPP in diet), Q (quadratic contrast of % SDPP in diet) and INT (interaction of parity and diet)

^d^ Included the covariance of total days the peripartum diet was fed in the mixed model for the All and Y sow group

^e^ Included the covariance of days from entry to weaning in the mixed model

### Blood sampling and analytical procedures

Two sampling times (2 days before expected parturition at 115 days of gestation and 4 days after parturition) were used for collection of blood from 57 randomly selected sows. Twenty-one sows were sampled twice and 36 once (either before or after parturition). Overall, a total of 78 samples were collected and 6 were discarded due to hemolysis. The final number of sows and analyzed samples per diet are presented in Table 7. Blood samples were collected from the tail vein using open bleeding into vacutainer tubes without anticoagulant. Blood samples were kept at ambient temperature for 30 to 45 min until clot formation, then subjected to centrifugation at 2000 g for 10 min. The serum was pipetted into new tubes, labeled by date and sow number, then stored at − 80 °C until analysis for cytokine and oxidation status parameters. Serum samples were analyzed by the Animal Nutrition and Welfare Service (SNIBA), Department of Animal Food Science Department, Universitat Autonóma de Barcelona.

Cytokine serum levels were determined using a cytokine and chemokine panel (ProcartaPlex Pig, Luminex B. V, Hertogenbosch, The Netherlands) as specified by the manufacturer: interferon alpha (IFN-α), interferon gamma (IFN-γ), tumor necrosis factor alpha (TNF-α), interleukin (IL), IL-1β, IL-4, IL-6, IL-8, IL-10 and IL-12. Readings were performed on the Luminex MAGPIX system (Luminex B. V, Hertogenbosch, The Netherlands).

Total antioxidant status (TAS), superoxide dismutase (SOD) and glutathione peroxidase (GPx) activities were determined using a kit from RANDOX laboratories (Crumlin, UK) and readings were performed using an AU400 analyzer (Beckman Coulter, Germany). Malonaldehyde (MDA) levels were determined using a TBARS Assay Kit (Cayman Chemical, Michigan, USA) following the manufacturer protocol and readings were performed using the Multiskan Sky (ThermoFisher Scientific, Waltham, MA, USA).

### Statistical analysis

All production data with sow as the experimental unit were statistically analyzed for the effects of sow parity, peripartum diet, and interaction of parity and peripartum diet (INT) using a mixed model (PROC MIXED) procedure of SAS (Statistical Analysis System, version 9.4, SAS Inst. Inc., Cary, NC). Linear (L) and quadratic (Q) contrasts were included in the model to test the significance of SDPP level used in the peripartum diet. A covariant was included in the mixed model for some variables to further evaluate the significance of diet or diet and parity interactions. The least squares mean (LSM) and pooled standard error of the mean (SEM) for diet effects are reported. In addition, Pearson correlation coefficients (r) and T-test probability for select variables were determined using the PROC CORR procedure of SAS.

Parity of sows used in this study ranged from 1 to 8 with parity 1 defined as a gilt having her first litter. However, parity distribution across dietary treatments was not evenly distributed, especially for parity 3 to 8 sows which represented only about 35% of the sows in the study (Table 1). Unequal parity distribution across dietary treatments can significantly skew calculated LSM when using sow parity in the model. Therefore, due to the unequal diet by parity distribution, parity 1 (*n* = 147) and parity 2 sows (*n* = 148 sows) were designated as parity group Y (*n* = 295) and parity 3 to 8 sows (*n* = 151 total sows) were designated as parity group M to increase the statistical power of a F-test to detect potential effects of diet, parity group, and interactions of diet and parity group on economically important variables such as litter size. A post hoc calculated sample size of 291 was estimated for detecting a 0.4 difference in a fixed effect using a power calculator (G*Power 3.1.2; https://gpower.software.informer.com/3.1/) for the above model of fixed effects and interactions using a F-test (*P* = 0.05). The designated parity Y or M group was used in the statistical model to test the significance of these designated parity groups, diet, and the interaction of designated parity group and diet for all sows. Furthermore, due to detection of the designated parity group and diet interactions for some of the litter size variables, datasets using only the group Y sows or the group M sows were analyzed separately so that the original parity of the sows within each subset of data could be evaluated using the mixed model with the actual parity, diet, and interaction of parity and diet.

In Tables 3, 4 and 5 production data results of all sows are listed under group All. Results of the combined parity 1 and 2 sows are listed under group Y with select variables also showing the LSM of the interaction of diet and parity by parity 1 (P1) or parity 2 (P2) sows. Results from the combined data of parity 3 to 8 sows are listed under group M. The covariance of total days the peripartum diets were fed was included in the model for average daily feed intake of the diets, and the days from entry in maternity to weaning was included in the model for backfat depth loss and backfat depth at weaning (Table 3). In Table 4, the model to evaluate total born litter size at the first parturition included the covariance of sow age at the initial parturition for the Y group of sows. Also, in Table 4 the total born litter size at the first parturition was included in the model for the number and percentage of stillborn and mummified pigs. The covariance of total born litter size of the first parturition was used in the model for total born in the second parturition, and the covariance of total born in the second parturition was included in the model for the number and percentage of stillborn and mummified pigs (Table 5). Pearson correlation coefficients for select litter size variables are presented in Table 6.

**Table 4:** Litter size during the first parturition by sow group and peripartum diet

		Peripartum diet, % SDPP^b^				Statistics (F-test, *P =*)^c^				
Variable	Group^a^	0	0.5	2.5	SEM	PR	D	L	Q	INT
Sows, n	All	147	167	138	–	–	–	–	–	–
	Y	94	111	90	–	–	–	–	–	–
	P1	47	55	45	–	–	–	–	–	–
	P2	47	56	45	–	–	–	–	–	–
	M	53	56	48	–	–	–	–	–	–
Total born pigs^d^	All	14.68	14.69	13.82	0.30	< 0.01	0.06	0.02	0.62	0.42
	Y	14.23	14.10	12.83	0.36	0.11	< 0.01	< 0.01	0.73	0.33
	P1	13.65	14.17	12.37	0.50					
	P2	14.81	14.02	13.29	0.50					
	M	15.10	15.29	15.00	0.64	0.59	0.93	0.82	0.78	0.42
Live born pigs	All	12.65	13.12	12.28	0.29	0.07	0.10	0.14	0.12	0.16
	Y	12.68	12.93	11.56	0.34	0.02	< 0.01	< 0.01	0.26	0.21
	P1	11.83	12.89	11.13	0.49					
	P2	13.53	12.96	11.98	0.49					
	M	12.76	13.37	13.13	0.58	0.41	0.72	0.80	0.43	0.55
Stillborn pigs^e^	All	1.84	1.40	1.34	0.17	< 0.01	0.06	0.08	0.09	0.71
	Y	1.41	1.05	1.16	0.18	0.18	0.22	0.49	0.10	0.25
	P1	1.72	1.16	1.09	0.24					
	P2	1.11	0.93	1.22	0.24					
	M	2.07	1.61	1.59	0.43	0.84	0.65	0.50	0.46	0.71
Stillborn, %^e^	All	11.78	8.77	9.29	1.01	0.03	0.06	0.23	0.04	0.46
	Y	9.98	7.04	9.04	1.14	0.20	0.14	0.98	0.04	0.12
	P1	12.25	8.19	8.05	1.61					
	P2	7.71	5.88	10.04	1.61					
	M	12.83	9.98	9.78	2.25	0.73	0.53	0.41	0.38	0.82
Mummies^e^	All	0.19	0.17	0.21	0.05	0.02	0.87	0.75	0.67	0.92
	Y	0.11	0.11	0.14	0.05	0.66	0.84	0.56	0.92	0.57
	M	0.26	0.31	0.28	0.14	0.29	0.96	0.99	0.77	0.54
Mummified, %^e^	All	1.22	1.11	1.46	0.39	0.07	0.80	0.56	0.74	0.94
	Y	0.75	0.68	1.18	0.44	0.44	0.66	0.40	0.76	0.30
	M	1.58	2.13	1.82	0.87	0.28	0.88	0.96	0.61	0.36

^a^ Results for group All included data of all sows, group Y included data only for young (parity 1 and parity 2) sows and group M included data only for mature sows (parity 3 to 8)

^b^ Values are least squares means (LSM) and pooled standard error of the mean (SEM) for peripartum diets by sow group. The interaction of diet and parity LSM and SEM are shown for some variables in the Y group as P1 (parity 1 sows) or P2 (parity 2 sows)

^c^ Each sow group was analyzed using a mixed model for the effects of PR (sow parity), D (peripartum diet), L (linear contrast of % SDPP in diet), Q (quadratic contrast of % SDPP in diet) and INT (interaction of parity and diet)

^d^ Included the covariance of sow age at the initial parturition in the mixed model used for Y group sows only

^e^ Included the covariance of total born pigs in the mixed model for each sow group

**Table 5:** Litter size of sows that farrowed a second litter by sow group and diet

		Peripartum diet, % SDPP^b^				Statistics (F-test, *P =*)^c^				
Variable	Group^a^	0	0.5	2.5	SEM	PR	D	L	Q	INT
Sows, n	All	138	155	133	–	–	–	–	–	–
	Y	87	103	85	–	–	–	–	–	–
	P1	45	50	43	–	–	–	–	–	–
	P2	42	53	42	–	–	–	–	–	–
	M	51	52	48	–	–	–	–	–	–
Total born pigs^d^	All	14.17	14.42	14.46	0.34	< 0.01	0.75	0.56	0.61	0.03
	Y	14.14	15.09	15.49	0.36	0.11	0.02	0.02	0.12	0.82
	P1	13.78	14.94	15.05	0.50					
	P2	14.50	15.25	15.93	0.51					
	M	14.46	13.50	13.32	0.56	< 0.01	0.26	0.19	0.25	0.50
Live born pigs	All	12.85	12.92	13.08	0.30	< 0.01	0.86	0.58	0.94	0.21
	Y	13.02	13.55	13.97	0.36	0.36	0.18	0.09	0.44	0.72
	P1	12.80	13.58	13.60	0.51					
	P2	13.24	13.53	14.33	0.52					
	M	13.05	11.99	11.83	0.55	< 0.01	0.20	0.16	0.20	0.79
Stillborn pigs^e^	All	0.96	1.24	0.98	0.13	0.19	0.09	0.55	0.03	0.54
	Y	0.78	1.17	1.00	0.13	0.17	0.06	0.53	0.02	0.84
	M	0.96	1.14	1.01	0.22	0.20	0.78	0.97	0.48	0.13
Stillborn, %^e^	All	6.81	8.05	6.93	0.74	0.16	0.40	0.75	0.19	0.87
	Y	6.06	7.83	7.09	0.98	0.37	0.32	0.69	0.14	0.57
	M	6.76	7.94	7.79	1.53	0.09	0.82	0.71	0.58	0.32
Mummified pigs^e^	All	0.44	0.40	0.49	0.05	0.66	0.44	0.33	0.41	0.14
	Y	0.37	0.40	0.54	0.06	0.26	0.09	0.03	0.99	0.42
	M	0.45	0.37	0.48	0.10	0.40	0.60	0.60	0.43	0.43
Mummified, %^e^	All	3.01	2.73	3.61	0.36	0.21	0.20	0.12	0.37	0.37
	Y	2.38	2.71	3.55	0.42	0.26	0.12	0.04	0.86	0.35
	M	3.10	2.64	4.24	0.80	0.54	0.22	0.15	0.45	0.31
Total born change^f^	All	−0.53	−0.30	0.61	0.42	< 0.01	0.13	0.04	0.99	0.05
	Y	−0.13	1.01	2.45	0.51	0.76	< 0.01	< 0.01	0.32	0.93
	P1	0.07	0.96	2.56	0.72					
	P2	−0.33	1.06	2.33	0.72					
	M	−0.70	− 2.03	−1.68	0.86	0.05	0.48	0.57	0.25	0.83
Live born change^f^	All	0.16	−0.31	0.68	0.41	< 0.01	0.22	0.18	0.26	0.06
	Y	0.26	0.66	2.17	0.50	0.26	0.02	< 0.01	0.96	0.79
	P1	0.84	0.88	2.33	0.71					
	P2	−0.33	0.45	2.02	0.71					
	M	0.27	−1.58	−1.31	0.82	0.04	0.19	0.30	0.11	0.95

^a^ Results for group All included data of all sows, group Y included data only for young (parity 1 and parity 2) sows and group M included data only for mature sows (parity 3 to 8). The interaction of diet and parity LSM and SEM are shown for some variables in the Y group as P1 (parity 1 sows) or P2 (parity 2 sows)

^b^ Values are least squares means (LSM) and pooled standard error of the mean (SEM) for peripartum diets by sow group

^c^ Each sow group was analyzed using a mixed model for the effects of PR (sow parity), D (peripartum diet), L (linear contrast of % SDPP in diet), Q (quadratic contrast of % SDPP in diet) and INT (interaction of parity and diet)

^d^ Included the covariance of total born pigs of the initial parturition in the mixed model used for each sow group

^e^ Included the covariance of total born pigs of the next parturition in the mixed model for each sow group

^f^ Total born change is total born litter size of subsequent parturition minus total born litter size of previous parturition. Live born change is live born litter size of subsequent parturition minus live born litter size of previous parturition

**Table 6:** Correlation coefficients for select variables related to litter size by sow group

	First parturition					Second parturition			
		Total born^a^					Total born^b^		
Variable	Group^c^	r	*P =*	n	Variable	Group	r	*P =*	n
Stillborn^a^, n	All	0.3698	< 0.0001	452	Stillborn^b^, n	All	0.1746	0.0003	426
	M	0.4577	< 0.0001	157		M	0.2739	0.0007	151
	Y	0.2746	< 0.0001	295		Y	0.1329	0.0276	275
Diet^d^	0	0.3020	0.0031	94	Diet^d^	0	−0.0296	0.7857	87
	0.5	0.3212	0.0006	111		0.5	0.2306	0.0191	103
	2.5	0.1881	0.0758	90		2.5	0.0842	0.4437	85
Sow age^a^	Y	0.0909	0.1216	295	Sow age^b^	Y	0.0914	0.1306	275
Diet^d^	0	0.1755	0.0906	94	Diet^d^	0	0.0893	0.4107	87
	0.5	−0.0013	0.9889	111		0.5	0.0572	0.5658	103
	2.5	0.1150	0.2806	90		2.5	0.1512	0.1673	85
Total born^b^	All	−0.0356	0.4635	426					
	M	−0.0020	0.9808	151					
	Y	−0.0129	0.8314	275					
Diet^d^	0	0.0194	0.8588	87					
	0.5	−0.1419	0.1527	103					
	2.5	0.1589	0.1462	85					

^a^ Designates variable of the first parturition

^b^ Designates variable of the second parturition

^c^ Values are Pearson correlation (r) coefficients, T-test probability (*P*) and number (n) of sows per groups All (all sows), Y (parity 1 and 2 sows) and M (parity 3 to 8 sows) during initial and next parturition

^d^ Values are r, *P*, and n by diet containing 0, 0.5 or 2.5% SDPP within the Y group of sows

Serology results were analyzed using a mixed model procedure (PROC MIXED) of SAS for the effects of sampling period (2 days before expected parturition at 115 days of gestation and 4 days after parturition), peripartum diet (0, 0.5 and 2.5% SDPP), and the interaction of sampling period and peripartum diet. The LSM and SEM for peripartum diet by sampling period for cytokine and oxidation status markers are presented in Table 7. Pearson correlation coefficients of serological variables with litter size variables are presented in Table 8.

**Table 7:** Serum cytokine and oxidation status of prepartum and postpartum sows fed peripartum diets with SDPP

		Peripartum diet, % SDPP^a^				Statistics (F-test, *P =*)^b^				
Variable	Period	0	0.5	2.5	SEM	SP	D	L	Q	INT
Sows, n^c^	−2 d	11	13	11	–	–	–	–	–	–
	+ 4 d	14	9	14	–	–	–	–	–	–
IFN-α, pg/mL	−2 d	0.97 (10)	0.49 (9)	0.58 (9)	0.38	0.80	0.17	0.30	0.14	0.88
	+ 4 d	1.27 (13)	0.45 (6)	0.55 (11)	0.47					
IFN-γ, pg/mL	−2 d	1.67 (10)	1.60 (9)	1.69 (9)	0.06	0.73	0.72	0.86	0.44	0.63
	+ 4 d	1.71 (13)	1.67 (6)	1.63 (12)	0.08					
IL-10, pg/mL	−2 d	3.16 (10)	3.43 (9)	3.45 (9)	1.79	0.25	0.70	0.52	0.63	0.81
	+ 4 d	3.61 (13)	5.51 (6)	5.95 (12)	2.20					
IL-1β, pg/mL	−2 d	82.7 (10)	24.5 (9)	8.98 (9)	45.2	0.88	0.23	0.18	0.35	0.99
	+ 4 d	82.1 (13)	31.6 (6)	18.9 (12)	55.4					
IL-4, pg/mL	−2 d	1.68 (10)	1.30 (9)	1.25 (9)	0.29	0.81	0.19	0.18	0.27	0.99
	+ 4 d	1.77 (13)	1.36 (6)	1.28 (12)	0.35					
IL-6, pg/mL	−2 d	32.5 (10)	15.6 (9)	8.46 (9)	14.1	0.81	0.24	0.25	0.26	0.91
	+ 4 d	28.5 (13)	7.36 (6)	12.6 (12)	17.2					
IL-8, pg/mL	−2 d	26.0 (10)	27.9 (9)	13.5 (9)	15.0	< 0.01	0.35	0.21	0.38	0.84
	+ 4 d	60.6 (13)	78.7 (6)	48.5 (12)	18.4					
TNF-α, pg/mL	−2 d	31.0 (10)	44.6 (9)	19.9 (9)	18.4	0.50	0.83	0.55	0.86	0.72
	+ 4 d	47.9 (13)	36.8 (6)	41.3 (12)	22.5					
IL-12, pg/mL	−2 d	146.2 (10)	89.9 (9)	63.9 (9)	33.5	0.91	0.15	0.22	0.17	0.59
	+ 4 d	122.6 (13)	69.4 (6)	98.9 (12)	41.1					
MDA, μM	−2 d	13.4 (11)	11.2 (13)	12.2 (11)	2.48	< 0.01	0.61	0.49	0.46	0.66
	+ 4 d	17.7 (12)	16.8 (8)	20.8 (13)	2.91					
SOD, U/mL	−2 d	0.97 (11)	0.62 (13)	0.75 (11)	0.15	0.07	0.12	0.20	0.11	0.75
	+ 4 d	1.17 (14)	0.97 (8)	0.87 (12)	0.18					
TAS, mmole/L	−2 d	0.47 (10)	0.61 (13)	0.65 (11)	0.07	0.57	0.15	0.13	0.23	0.66
	+ 4 d	0.56 (11)	0.63 (6)	0.63 (13)	0.09					
GPx, U/L	−2 d	7382 (11)	8188 (13)	9055 (11)	496	0.92	0.01	< 0.01	0.28	0.87
	+ 4 d	7599 (14)	8340 (9)	8806 (14)	548					

^a^ Values are least squares diet means, pooled standard error of the mean (SEM) and number (n) of serum samples analyzed by sampling period, 2 d before expected parturition at day 115 of gestation (−2 d) and 4 d after parturition (+ 4 d)

^b^ Probability values for SP (sampling period), D (diet), L (linear contrast of % SDPP in diet), Q (quadratic contrast of % SDPP in diet), and INT (interaction of SP and D)

^c^ Number of sows sampled by period and diet; number of samples analyzed per cytokine or oxidation status marker varied due to discarded samples affected by hemolysis

**Table 8:** Correlation coefficients of serology variables with litter size variables

First parturition				Second parturition			
	Stillborn^a^				Total born^b^		
Variable	r	*P =*	n	Variable	r	*P =*	n
Total born^a^	0.4874	0.0003	50	Total born^a^	−0.0078	0.9584	47
IFN-α^a^	−0.1202	0.5503	27	IFN-α^b^	0.0236	0.9033	29
IFN-γ	−0.2280	0.2528	27	IFN-γ	−0.2736	0.1436	30
IL-10	0.1724	0.3898	27	IL-10	0.1549	0.4136	30
IL-1β	−0.1251	0.5340	27	IL-1β	0.0447	0.8144	30
IL-4	−0.0488	0.8090	27	IL-4	0.1746	0.3561	30
IL-6	0.0919	0.6486	27	IL-6	0.0207	0.9135	30
IL-8	−0.0488	0.8395	27	IL-8	0.1175	0.5363	30
TNF-α	0.4123	0.0326	27	TNF-α	0.0318	0.8675	30
IL-12	−0.0764	0.7050	27	IL-12	0.0719	0.7059	30
MDA	−0.0276	0.8770	34	MDA	−0.0539	0.7734	31
SOD	−0.0154	0.9311	34	SOD	0.0451	0.8062	32
TAS	0.1148	0.5249	33	TAS	0.0125	0.9498	28
GPx	0.1769	0.3169	34	GPx	−0.3581	0.0347	35

Values are Pearson correlation (r) coefficients, T-test probability (*P =*) and number (n) of observations per variable

^a^ Total born or stillborn results of first parturition were correlated with serology variables of sows sampled 2 days prepartum

^b^ Total born results of second parturition were correlated with serology variables of sows sampled 4 days postpartum

Differences in results were considered significant at *P* < 0.05 and a tendency for differences in results are reported for probabilities between *P* > 0.05 and *P* < 0.10.

## Results

### Production data results

Results of peripartum feed intake, sow backfat depth and postweaning sow performance variables by peripartum diet and sow group are presented in Table 3. Using all sows, the average days from entry in maternity to the day of the initial parturition differed for the effect of parity and the interaction of diet and parity, with a quadratic tendency (*P* = 0.06) for SDPP level in the diet. In the Y group, entry to farrow days was significant for diet and increased linearly with level of SDPP in the diet. For the M group the effect of diet was significant and tended (*P* = 0.06) to increase linearly with SDPP level in the diet. The average days of the peripartum feeding period for the All, Y and M sow groups presented the same parity, diet, or parity and diet interaction significant differences or trends as for the average days from entry to farrowing.

The covariance of total peripartum feed days was included in the model for each sow group to evaluate differences in average daily peripartum feed intake corrected for feed refusals. For all sows, parity differed, but there were no significant effects of diet or interaction of diet and parity for peripartum feed intake. There were no differences of parity, diet, or the interaction of parity and diet for the Y group. When using total peripartum feed days in the model for the M sow group, the convergence criteria for this covariant could not be met, therefore it was excluded from the model to evaluate feed intake for the mature sow group. Feed intake for the M group of sows was significant for diet with a quadratic tendency (*P* = 0.06) for SDPP level with 0.5% SDPP having the lower feed intake.

Average backfat depth at weaning and backfat loss from entry to weaning were evaluated with the covariance of days from entry in maternity to weaning (30.42 ± 0.09) in the model (Table 3). There were no significant effects of diet or interaction of parity and diet for average backfat depth at entry, at weaning, or for backfat loss from entry to weaning for each of the sow groups, with the exception that there was a downward quadratic trend (*P* = 0.09) for sows fed 0.5% SDPP to have a lower backfat depth at weaning in the All sow group. The effect of parity differed (*P* <  0.05) for backfat depth at entry for the Y group and differed for backfat depth at weaning and for backfat loss from entry to weaning for the All and Y group of sows.

During the initial parturition which used 452 sows, 20 died and 5 were culled postweaning. There were no significant effects of parity or diet on the percentage of sows that were moved to breeding for any of the sow groups. One serviced sow previously fed the peripartum diet with 0% SDPP in the first parturition failed to farrow the next litter.

Average wean-to-first-estrus interval and average wean-to-final-service interval did not significantly differ by diet or parity for the All sow group, however there was a significant parity by diet interaction for wean-to-first-estrus interval for the M sow group and a significant parity by diet interaction for wean-to-final-service interval for the Y sow group.

The results for litter size variables during the first parturition by sow group and peripartum diet are presented in Table 4. When analyzing data from all sows, there was a significant effect of parity and a significant linear effect of diet for total born pigs per litter (sum of stillborn, mummified, and live born pigs) for sows allotted to the 2.5% SDPP diet, which had the lowest number of total born pigs per litter. The linear effect of SDPP level in the peripartum diet for total born pigs per litter was also significant for the Y group of sows but did not differ for the M group of sows. The farm was able to provide records for the age of the parity 1 and 2 sows at the initial parturition to evaluate if sow age was a factor associated with the variable total born litter size by allotted diet. Average sow age at the initial parturition for parity 1 sows was 51.5, 51.1 and 51.0 weeks and 72.1, 71.8 and 71.8 weeks for parity 2 sows per respective increasing level of SDPP in the diet. The total born litter size for the Y group of sows was evaluated using the covariance of sow age in the model, however sow age did not change the significance of the differences for the diet effect. The correlation coefficient (r = 0.0909) was low between sow age and total born litter size of the Y sow group (Table 6).

There was a tendency (*P* = 0.07) for an effect of parity on live born pigs for the All sow group. Live born pigs per litter in the Y group was significant by parity, diet, and presented a linear reduction in litter size as SDPP level increased. There were no significant effects of parity or diet for live born pigs in the M sow group.

The model used to analyze the number of stillborn and mummified pigs included total born pigs per litter due to the allotment differences in total born by diet. The number of stillborn pigs per litter differed by parity with a downward linear tendency (*P* = 0.06) as SDPP level increased in peripartum diets for the all sow group. There were no significant parity or diet effects on number of stillborn pigs for Y or M group sows, however there was a tendency (*P* = 0.10) for a downward quadratic response to increasing dietary SDPP in the Y group of sows. Within the Y group of sows there was an indication (*P* = 0.12) of a parity and diet interaction; parity 1 sows presented a pattern of decline in number of stillborn pigs with increasing SDPP level, while parity 2 sows presented a quadratic pattern to SDPP level with parity 2 sows fed 0.5% SDPP having the lowest average number of stillborn pigs.

When stillborn pigs were expressed as a percentage of total born pigs in the All sow group, there were significant effects of parity, and a quadratic reduction of percent stillborn pigs as SDPP level increased. Similarly, for the Y sow group there was a significant quadratic reduction of percent stillborn pigs as SDPP level increased and an indication (*P* = 0.12) of a parity and diet interaction like that for number of stillborn pigs. For the M sow group there were no significant effects of parity or diet for percentage of stillborn pigs.

Mummified pigs per litter significantly differed by parity and mummified pigs as a percentage of total born tended (*P* = 0.07) to differ by parity for the All sow group, however there were no significant effects of parity or diet for number or percentage of mummified pigs per litter for Y or M group sows.

Results of litter size parameters in the second parturition are presented in Table 5. The total born pigs in the next litter were analyzed with the covariance of total born pigs in the first parturition. Total born pigs per litter in the second parturition for the All sow group was significantly affected by parity and there was a significant parity by diet interaction. Total born pigs in the next litter was increased linearly (*P* = 0.02) for Y sows fed increased SDPP levels in the peripartum diet that was only fed during the first parturition, but there was no significant parity and diet interaction. Both parity 1 and parity 2 sows presented a similar linear increase in total born pigs as SDPP level increased in the diet. Sow age at the second parturition was also evaluated as a covariant in the model for the Y sow group. Average sow age at the next parturition for parity 1 sows was 73.9, 72.6 and 72.4 weeks per respective increasing level of SDPP and for parity 2 was 93.4, 93.5 and 93.0 weeks. However, sow age did not have any significant influence on the effect of diet for total born pigs. Sow age at the second parturition had a low correlation (r = 0.0914) with total born litter size (Table 6). Total born pigs per litter in the second parturition for the M sow group was significantly affected by parity but was not significantly affected by diet.

Live born pigs per litter in the second parturition for all sows and M sow groups were significantly affected by parity, but not by diet. Live born pigs in the second parturition for the Y sow group tended (*P* = 0.09) to present a linear increase in litter size relative to increased SDPP level fed in the first parturition.

The covariance of total born litter size in the second parturition was used in the model to evaluate the number and percentage of stillborn and mummified pigs in the second parturition. Parity or parity by diet interactions were not significant for number or percentage of stillborn or mummified pigs per litter in the second parturition for any sow group, except for a tendency (*P* = 0.09) for a parity effect on percentage stillborn pigs in the M group of sows. However, number of stillborn pigs increased quadratically with SDPP level for all sows and the Y sow group. There were no parity or parity and diet interaction for the number or percentage of mummified pigs for any of the sow groups. In the Y sow group there was a significant linear increase in number and percentage of mummified pigs with increased SDPP level in the peripartum diet.

The change in total and live born pigs per litter from the previous to the next parturition was calculated considering that litter size for total born in the previous parturition was already established before the peripartum diet was fed, but that subsequent litter size could potentially be impacted by feeding SDPP in the peripartum diet. The change in total born pigs per litter for all sows was significant for parity and the interaction of parity and diet and presented a linear increase (*P* <  0.05) with increasing SDPP level in the peripartum diet. Change in total born for the Y sow group was linearly increased (*P* <  0.01) with increased SDPP level fed during the first parturition. Within the Y group of sows, the linear pattern of increased change in total born was similar for parity 1 and parity 2 sows and there was no significant parity and diet interaction. There were no significant diet effects on change of total born for the M sow group, but the effect of parity differed.

Change in live born litter size was significantly affected by parity for all sows and the M sow group and there was a parity by diet interaction for all sows. For the Y sow group there was a linear increase (*P* <  0.01) in change of live born pigs as SDPP level increased in the diet, whereas there were no significant effects of diet in the M sow group. Within the Y group there was no significant diet and parity interaction. Both parity 1 and parity 2 sows presented an increased linear pattern of change in live born pigs as SDPP level increased in the peripartum diet.

Pearson correlation coefficients and probability (T-test) for variables related to litter size in the first and second parturition are presented by sow group in Table 6. In the first parturition, the number of stillborn pigs had a significant and positive correlation with total born pigs and the correlation was higher for the M group (r = 0.4577) relative to the Y group (r = 0.2746). Sow age at the first parturition had a low correlation (r = 0.0909) with total born in the Y sow group. The correlation of total born pigs in the first parturition with total born in the second parturition was not significant and was slightly negative for each sow group. In the second parturition, the number of stillborn was significant and positively correlated with total born pigs, again with a higher coefficient for the M group (r = 0.2739) relative to the Y group (r = 0.1329). As in the first parturition, sow age at the second parturition for the Y group of sows had a low correlation (r = 0.0914) with total born pigs in the second parturition.

### Serology results

Serology results presented in Table 7 revealed no significant effect of diet on the various cytokines analyzed. Higher levels of IL-8 were detected (*P* <  0.05) during the postpartum period compared to the prepartum period. There was a linear increase (*P* <  0.01) for GPx as SDPP in peripartum feed increased. The other oxidation status markers were not different among peripartum diets, but sampling period differed (*P* <  0.05) for MDA and tended (*P* = 0.07) to differ for SOD, both of which were higher in the postpartum period (day 4 of lactation) compared to the prepartum period (2 days before expected parturition at 115 days of gestation). There were no significant interaction of diet and sampling period for cytokine or oxidation status markers.

Pearson correlation coefficients of prepartum serology variables with stillborn pigs in the first parturition and postpartum serology variables with total born pigs in the second parturition are presented in Table 8. The number of stillborn pigs in the first parturition were significantly and positively correlated (r = 0.4874) with total born pigs and with serum TNF-α (r = 0.4123) samples collected prepartum. In the second parturition there was a significant negative correlation (r = − 0.3581) between total born pigs and results of GPx samples collected on day 4 postpartum.

## Discussion

Previous studies using 0.5% SDP in lactation feed have reported several production improvements including higher ad libitum lactation feed intake by parity 1 and 2 sows, reduced wean-to-first-estrus interval for parity 1 sows [20], increased subsequent farrowing rate [21], increased pre-wean survival [19, 22, 23], increased litter weight, and increased average pig weight at weaning [20, 21] with more full-value pigs weaned per litter [20, 21]. However, data is lacking to establish the optimum level and feeding duration of SDP in sow diets at strategic periods of the breeding herd lifecycle, especially when using current sow genetics that have been selected to produce larger litter size than those of the past decade. The current study is the first known to evaluate the short-term feeding of variable levels of SDPP in peripartum feed for sows when provided from entry in maternity (109 days of gestation) through day 5 postpartum.

Nutrition studies conducted at commercial farms can provide relevant data with large numbers of sows per diet, but results can sometimes be challenging to interpret. Appropriate statistical models that adjust for variance associated with physiological, production, and management factors are needed to best interpret effects of dietary treatments. Parity of sow is an important fixed effect to evaluate in sow studies because physiological variables, such as sow body weight, pig weight at birth or at weaning, and total born litter size are known to differ by parity of the sow, particularly for primiparous sows compared to multiparous sows. When using a rolling allotment of diets to sows by parity over a 4-month interval under a continuous farrowing schedule, it becomes difficult to maintain a balanced allotment of diets to sows by parity. The number of mature (parity 3 to 8) sows allotted across dietary treatments in the current study was variable and collectively represented 35% of the sows used in the study (Table 1). However, the number of young (parity 1 and 2) sows which collectively represented 65% of the sows used in the study, were equally allotted by parity 1 or 2 sows within diet but there were more young sows allotted to the 0.5% SDPP diet compared to the other diets. When using parity of sow in the statistical model, the calculated LSM and SEM can become skewed especially with unequal distribution of sows allotted to diet by parity and the effects of dietary treatments may become less detectable. Therefore, data from sows were grouped as Y (combined parity 1 and 2 sows) or M (parity 3 to 8 sows) and then analyzed as separate datasets to reduce the skewed effects on LSM and SEM when using the unequal distribution of parity by diet in the model analysis for all sows.

The results for variables associated with feed intake of the peripartum diets, backfat depth of sows, and postweaning sow reproduction are presented in Table 3. By design, the peripartum diets were provided to sows at 3 kg per day from entry in maternity (approximately day 109 of gestation) to the day of parturition, then sows were provided 4.5 kg of the peripartum diet on day 1 to 5 postpartum. Daily feed refusals of the peripartum diets were recorded and average daily feed intake of the peripartum diets were corrected for feed refusals. Feed refusals by individual sows can be common, especially around the time of parturition. Due to the variable days from entry to parturition, the covariance of total days the peripartum diets were fed was used in the model to evaluate average daily peripartum feed intake for datasets including all sows and Y sows, but could not be used to evaluate feed intake of sows in the M group because convergence criterion for this covariant was not met and LSM for diet could not be calculated. Average daily peripartum feed intake corrected for feed refusals and adjusted by total peripartum feed days did not differ for the effect of diet for all sows or the Y sow group. Without using the covariance of total peripartum feed days in the M group of sows, the effect of peripartum diet presented a significant quadratic response to SDPP level in the diet, however only 4 mature sows had any recorded feed refusals so the effect of diet was likely related to differences in total peripartum feed days.

After ceasing the feeding of the peripartum diets at day 5 postpartum, sows were provided a common lactation feed ad libitum (3 feedings per day) to weaning and a common gestation feed to the next parturition. Average sow backfat depth at entry and weaning was recorded and results indicated no significant effects of peripartum diet on backfat loss from entry in maternity to weaning. Days from entry to weaning (average 30.42 days) was used as a covariant in the model for evaluating backfat depth at weaning and backfat depth loss, but this covariant did not change the effects of diet on these variables. These results suggest that variations in feed refusals of the peripartum diets had limited impact on sow backfat loss from entry to weaning. However, the role of the common lactation feed consumption on backfat loss is unknown because feed consumption of the common lactation diet was not recorded. In a previous publication [20] the use of 0.25% SDP in lactation feed for 14 days for sows farrowed in summer months reduced (*P* = 0.06) sow body weight loss of parity 1 and 2 sows and increased (*P* <  0.01) lactation feed intake, whereas in winter months there was a tendency (*P* = 0.09) for reduced sow body weight loss during a 14 d lactation period with no significant effect on sow feed intake when primiparous and multiparous sows were fed 0.25% SDP in lactation feed.

Lactation feed intake of parity 1 and 2 sows during summer months has also been shown to significantly increase with 0.5% SDP in sow diets fed during an 18 day lactation with a reduced wean-to-first-estrus interval for parity 1 sows [20]. However, diet effects on the wean-to-first-estrus interval and wean-to-final-service interval for sows fed the peripartum diets (Table 3) was not significantly different for all sows or the Y or M sow groups when analyzed with or without the covariance of the interval of days from entry in maternity to weaning (average 30.42 days). The effects of dietary SDP on wean-to-first-estrus interval may differ based on dietary level and duration of the lactation period, along with seasonal effects on sow feed intake during lactation.

The total born litter size at the first parturition (Table 4) was significantly lower for sows allotted to the 2.5% SDPP diet. Although total born litter size had been established before sows were provided the peripartum diets, the difference in total born by diet at allotment made it more difficult to interpret diet effects on other litter size variables. Age of sow at the first parturition was available for parity 1 and 2 sows and was used as a potential covariant in the model to explain the lower total born litter size of the parity 1 and 2 sows allotted to the 2.5% SDPP diet. Correlation of sow age and total born was very low (Table 6) and the covariance of sow age did not change the effect of diet on total born. Sow age of the Y sow group at the second parturition also had a very low correlation with total born of the second parturition and did not change the effect of diet on total born in the second parturition when used in the model.

In the Y group of sows there was a linear decrease in live born as SDPP level increased, but there was an indication (*P* = 0.12) of a quadratic effect of SDPP for live born pigs from all sows during the first parturition. Obviously, the difference in live born litter size relative to total born is the sum of stillborn and mummified pigs. Stillborn pigs per litter was moderately correlated with total born litter size in the first parturition, but more so in the M group (r = 0.4577) compared to the Y group (r = 0.2746) of sows (Table 6). Using total born litter size as a covariant in the model, the number of stillborn pigs from all sows had a tendency (*P* = 0.06) for a diet effect with a linear decline in stillborn as level of SDPP increased. Although diet effects on stillborn pigs in the first parturition were not significant for the Y or M group of sows, the numerical indication followed a similar pattern as for all sows with less stillborn pigs per litter for sows fed diets with SDPP. For stillborn pigs as a percentage of total born pigs there was a significant downward quadratic (*P* = 0.04) response as level of SDPP increased for all sows and the Y group of sows. Other factors such as litter size, birth weight, sow weight, parity, and assisted births can also impact stillbirths [12], but in our study total born litter size as a covariant in the model did not change the significance of the diet effect.

The downward quadratic response to SDPP level for percentage of stillborn pigs could reflect a dietary response because sows had consumed the peripartum diets for an average of 6.1 days prepartum. Other studies have demonstrated reduced parturition time and stillbirths are associated with a higher energy status of the sow [32] and use of fiber in sow diets [33]. However, stillbirths were not affected by increased feeding levels in late gestation [4], but gilts with lower feed intake had a lower percentage of stillbirths than gilts fed higher feed intake in late gestation [5]. Stillbirths occur during parturition and are highly associated with a prolonged parturition time [16]. In our study, parturition duration was not recorded, but future studies should be designed to understand if dietary SDPP affects duration of parturition.

In the second parturition (Table 5) there was a significant linear increase in total born litter size and a similar tendency (*P* = 0.09) for a linear increase in live born litter size for the Y group of sows that had been fed increasing SDPP levels in peripartum feed during the first parturition. The covariance of total born litter size in the first parturition was used in the model for analysis of total born litter size in the second parturition because of the allotted diet differences for total born in the first parturition. However, correlations of total born in the first parturition with the second parturition total born litter size were very low within each sow group (Table 6) and did not significantly influence the diet effect of total born litter size in the second parturition. Within the Y sow group, the linear pattern of response to litter size and change in litter size was consistent for both parity 1 and parity 2 sows. The change in litter size from the first parturition to the second parturiton for the Y group of sows resulted in approximately 1 and 2.45 more change in total born pigs per litter for the sows fed peripartum diets with 0.5 and 2.5% SDPP, respectively. In a past study [20] there was a numerical indication (*P* = 0.22) for about 0.9 more live pigs born per litter for gilts in their next parturition when previously fed 0.5% SDP in lactation feed during their initial lactation, which averaged about 18 days. At the commercial farm for the current study, the average total born litter size during the previous year of the study reported a 0.4 pig increase from parity 1 to 2, a 0.2 pig increase from parity 2 to 3, and a 0.3 pig increase from parity 3 to 4, then the average total born declined as parity increased beyond parity 4. During the previous year before the current study was initiated, the farm was transitioning to a different genetic supplier so the production records of total born litter size changes by parity may not reflect differences observed in the current study which only used sows from the current genetic supplier (Naima Choice Genetics). Although it had been over 4 months since SDPP had been fed in the current study, prior feeding of SDPP during the initial 5 days postpartum of the previous lactation could have potentially impacted postpartum uterus recovery and possibly affected ovarian function. Small follicles (< 5 mm) are present in the ovaries of lactating sows and they undergo atresia or recruitment for further development in later lactation prior to ovulation 4 to 7 days post-weaning [34].

Reproductive performance can vary by individual sow within the same farm, especially in hot climates [35]. However, the number of pigs born alive per litter for parity 1 and 2 sows has been considered a good predictor for lifetime sow reproductive performance [36, 37]. Young parity sows with low litter size are often culled, while those with higher litter size are kept in the herd. In our study, the linear increase in total and live born litter size of the next parturition for the young parity sows previously fed increased levels of SDPP during their initial parturition suggest that SDPP in peripartum feed can benefit subsequent litter size and potentially reduce the culling rate of young parity sows due to low litter size.

The potential mechanisms of SDPP in peripartum diets that resulted in reduced stillborn pigs in the first parturition and the longer-term increase in litter size in the second parturition for young parity sows fed peripartum diets containing SDPP are not clearly understood. In other studies, during late pregnancy under LPS-induced inflammation, mice fed diets with 8% SDP had reduced overstimulation of pro-inflammatory cytokines in uterine mucosa and placenta and had lower lethargic reactions to LPS [29]. Recently, sows and their weaned progeny had lower serum TNF-α and cortisol when the sows were fed 1% SDP in feed during the last trimester of gestation and during lactation compared to control sows and their progeny [38, 39]. However, in the current study there were no significant diet effects related to serum cytokine profiles of peripartum sows (Table 7) before or after parturition. Postpartum dysgalactic syndrome sows have more pronounced and significant changes in some hormone, metabolic and inflammatory serum markers than normal sows before and after parturition [14, 15]. The lack of detectable diet differences in our study for cytokine profiles may have been due to variable stress status of the sampled sows near parturition or insufficient sample size. A post hoc power test for a significant F-test probability with a model of fixed effects and interactions indicated 126 samples were needed to detect a 0.4 difference of the fixed effects. In Table 8 there was a significant moderate correlation (r = 0.4123) of prepartum TNF-α and number of stillborn in the first parturition suggesting a potential correlation of this inflammatory cytokine with increased stillborn pigs. When postpartum cytokines were correlated with total born in the second parturition, there were no significant correlations but an indication (*P* = 0.1436) of an inverse correlation (− 0.2736) with IFN-γ. For oxidation status markers, there was a linear increase in serum GPx as dietary SDPP level increased in both sampling periods suggesting an improved antioxidant status of sows fed SDPP around parturition (Table 7). However correlation of prepartum GPx with number of stillborn in the first parturition was not significant and low (r = 0.1769), while postpartum GPx had a significant and moderate inverse correlation (r = − 0.3581) with total born litter size in the second parturition. However, these correlations of serological markers with stillborn pigs and litter size include only a subset of 50 or less sows used in the study.

## Conclusions

Strategic use of SDPP in peripartum sow feed may have merit for reducing stillborn pigs and have longer-term beneficial effects on litter size in the next parturition for parity 1 and parity 2 sows.

## Data Availability

The data that supports the findings of this study are available from the corresponding author upon reasonable request.

## Abbreviations

SDP: Spray-dried animal plasma
SDPP: Spray dried porcine plasma
SDBP: Spray-dried bovine plasma
GPx: Glutathione peroxidase
SOD: Superoxide dismutase
MDA: Malonaldehyde
TAS: Total antioxidant status
TNF-α: Tumor necrosis factor alpha
IFN-α: Interferon alpha
IFN-γ: Interferon gamma
IL-1β: Interleukin 1β
IL-4: Interleukin 4
IL-6: Interleukin 6
IL-8: Interleukin 8
IL-10: Interleukin 10
IL-12: Interleukin 12
SAS: Statistical Analysis System
PROC MIXED: Mixed model procedure of SAS
PROC CORR: Procedure of SAS for calculating Pearson correlation coefficients
LSM: Least squares mean for peripartum diet
SEM: Pooled standard error of the mean for peripartum diet
r: Pearson correlation coefficient
*P*: Probability of F-test or T-test
L: Linear contrast of peripartum diet containing 0, 0.5 or 2.5% SDPP
Q: Quadratic contrast of peripartum diet containing 0, 0.5 or 2.5% SDPP
PR: Fixed effect of sow parity or designated sow parity
D: Fixed effect of peripartum diet
INT: Interaction of PR or SP and D
All: Dataset analyzed that included all parity sows
Y: Dataset analyzed using only young sows (combined data of parity 1 and parity 2 sows)
P1: Parity 1 sows (intended as gilt having first litter)
P2: Parity 2 sows
VTM: Vitamin and trace mineral premix
